# Interdisciplinary Research Maps: A new technique for visualizing research topics

**DOI:** 10.1371/journal.pone.0242283

**Published:** 2020-11-24

**Authors:** Mauricio Marrone, Martina K. Linnenluecke

**Affiliations:** 1 Department of Accounting and Corporate Governance, Macquarie Business School, Macquarie University, Sydney, New South Wales, Australia; 2 Centre for Corporate Sustainability and Environmental Finance, Macquarie Business School, Macquarie University, Sydney, New South Wales, Australia; Shantou University, Guangdong, CHINA

## Abstract

This article introduces Interdisciplinary Research Maps as a novel visualization technique to assist with interdisciplinary research analytics and to map common (and distinct) topics across publications from different disciplines. We detail the method for this technique which is based on entity linking and illustrate its application to a sample of articles sourced from the top business/management and environmental sciences journals. Both fields have separately been criticized for a lack of interdisciplinary research to co-create insights for tackling pressing environmental issues such as climate change. Our mapping approach provides a starting point for exploring similarities and differences in research topics across these fields. The mapping technique introduced here has broader applicability to facilitate the creation and exchange of knowledge across fields. We discuss avenues for visualization techniques to bridge the different fields by focusing on identifying common concepts to provide a basis for future analysis.

## 1 Introduction

The premise that interdisciplinary research can tackle pressing environmental issues such as climate change holds forth great promise–the implicit assumption is that interdisciplinary efforts can overcome limited and narrow disciplinary understandings of solutions and contribute to better problem-solving efforts. However, the sheer magnitude of information and knowledge embedded in individual disciplines, the lack of a common shared understanding of disciplinary knowledge, differences in research interest, methods, data, analytical approaches, as well as the complexity of coordinating research amongst researchers from different disciplines are still limiting factors [1, 2]. Tools for evidence synthesis are beginning to emerge, but are not yet commonly used [3], especially for interdisciplinary mapping reviews [4, 5].

To advance interdisciplinary research endeavors, our objective is to provide a new visualization technique to identify and display common (and distinct) topics in interdisciplinary research efforts. Specifically, we introduce Interdisciplinary Research Maps as a novel visualization technique to assist with interdisciplinary research analytics across publications from different disciplines. We discuss how Interdisciplinary Research Maps can be applied to bridge disciplinary areas by mapping and visualizing where disciplines have separate concepts, ideas and topics, where boundaries separating disciplines might be dissolving, and where there might be possibilities for new hybrid fields to emerge [6]. Prior research has shown that a focus on shared concepts, ideas and topics across disciplines can greatly help researchers to improve collaboration in interdisciplinary research [1].

The Interdisciplinary Research Maps technique builds on entity linking. Entity linking (further detailed in section 3.2) is based on the use of algorithmic technology that allows for the identification of meaningful concepts and text sequences (here referred to as ‘topics’) in unstructured text, and thus for the identification of common topics from the articles included for analysis. Here, we illustrate the application of Interdisciplinary Research Maps to a sample of articles sourced from the top business/management and environmental sciences journals to provide a starting point for exploring similarities and differences in research topics across fields.

The article seeks to contribute to the mapping of scientific discourses by bringing together knowledge from across disciplines to inform research debates. We offer a method that allows for an unbiased synthesis of available evidence to contribute to dialogue across different research communities. The is structured as follows. First, we provide a brief background on mapping and visualizing academic discourses [7]. Next, we detail our new method and show its application to our sample data to create an interdisciplinary research map. The technique developed here has broader applicability to facilitate the creation and exchange of knowledge across fields. We discuss avenues for Interdisciplinary Research Maps to bridge different fields by focusing on defining common concepts and to provide a basis for future analysis.

## 2 Mapping academic discourses

Researchers have developed a variety of different approaches for mapping and visualizing academic discourses. Many of these approaches focus on the analysis of academic publications within a particular field (often based on topic modelling or topic synthesis) [8], however, there are still few studies on the analysis of interdisciplinary academic discourses. Van Leeuwen and Tijssen [9], for instance, study the extent to which disciplines are interrelated through citation links, analyzing journal-to-journal citation data from the Journal Citation Report database. However, the authors note that their analysis provides limited insights in terms of mapping interdisciplinary connections between scientific areas. Other authors have focused on analyzing the content of grant proposals in order to document interdisciplinary research endeavors [10]. A substantial effort has also gone into identifying cross-citation networks across fields [11], however, the outputs generated are typically in the form of citation networks, with limited insights into topic overlaps.

The approach introduced here offers an improved approach to topic modeling. Topic modelling typically focuses on identifying a group of words (i.e., topic) from a collection of textual data [12–14], but has a number of limitations. In topic modelling, the user establishes the number of topics that will be extracted, which is based on an arbitrary selection [15]. Second, the researcher needs to label the correlated groups of terms that are generated by the statistical algorithms underlying the method [16], which is also based on user preferences. The labeling of topics is usually undertaken based on the most frequently occurring words in a group of terms, but word frequency and probability distributions do not provide a straightforward basis for naming a topic [17]. To carry out topic modelling, a researcher therefore needs to have a good understanding of the text corpora being analyzed and/or requires access to experts who can assist with this process [17, 18]. Given that the researcher is largely responsible for deciding on the extraction and labelling of topics, topic modelling approaches can therefore be biased and limited in their usefulness [18].

In the next section we detail our approach which is an automated approach to a mapping review and can therefore overcome user limitations associated with manual decisions in topic extraction and labelling [12, 13]. Furthermore, the approach is applicable to textual data from different disciplines, and therefore suitable for the development of Interdisciplinary Research Maps.

## 3 Method

**Fig 1:**
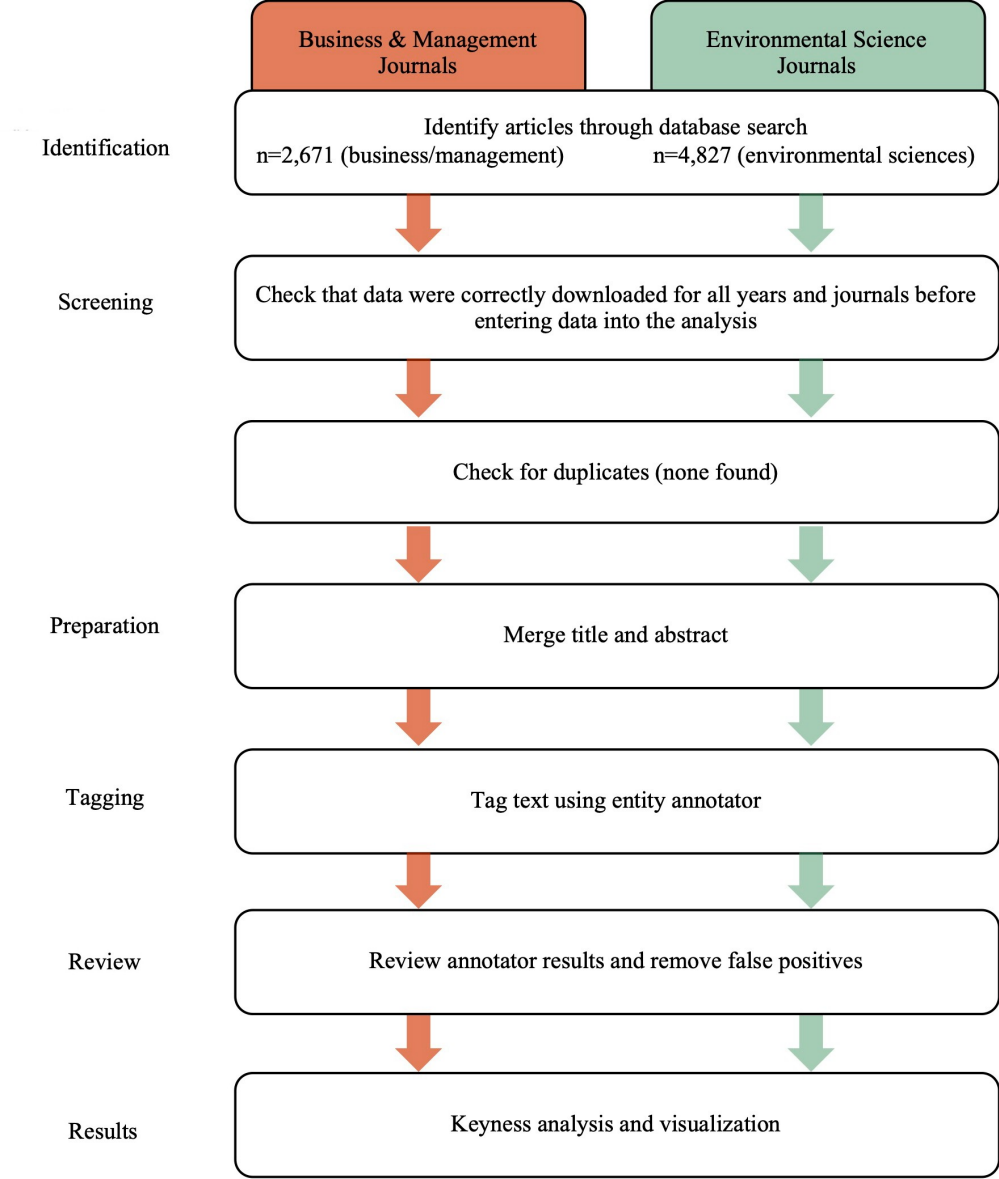
Flow chart.

The method introduced here involves several different steps (see also Fig 1), detailed in this section. First, we selected a sample of articles to demonstrate the visualization technique in this study. We sourced data through the Scopus database, selecting articles published in the top four journals by impact factor in the areas of *business/management* and *environmental sciences* (published between 2011-01-01 and 2020-09-24) and downloaded information (abstracts, titles, publication years). The following journals were included for the area of business/management: The *Academy of Management Review*, the *Journal of Management*, the *Academy of Management Journal*, and the *Journal of International Business Studies*. In addition, the following journals were included for the area of environmental science: *Nature Climate Change*, Global Environmental Change, *Annual Review of Environment and Resources*, and *Wiley Interdisciplinary Reviews Climate Change*. We decided not to include the journal Tourism Management due to its narrower focus on the planning and management of travel and tourism. We selected 2011-01-01 as the starting date as this provided a common timeframe across all journals. We recognize that a focus on high impact factors has limitations; however, we see the approach here as a first attempt to visualize different knowledge domains which can be expanded and refined in future research.

In a next step, we downloaded associated journal citation records from the Scopus database which fully indexes all eight journals, and extracted the abstracts, titles and years for further analysis. We decided to only use the titles and abstracts for our analysis for a number of reasons: (1) The decision to include only the academic abstracts rather than the entire articles was based on the view that such abstracts are likely to contain the most important aspects of the articles they describe [19]. Abstracts of academic articles are typically very concise and contain an overview of the core topics contained in the associated articles. More specifically, when comparing the usefulness of abstracts and full-length articles for textual analysis, Crawford et al. [19] were unable to find a clear advantage of using the full-length versions. (2) We decided not to use author keywords as prior research has raised several issues regarding the use of author keywords in entity linking. Entity linking works best with surrounding text as context [20], but authors sometimes only use acronyms as keywords, and provide a very limited number of 3–5 words that are at times not reflective of the article. However, it is certainly possible to just use the full text of articles or author keywords with the technique presented here, and other researchers using our proposed technique would be able to make this decision based on their own preferences.

We manually inspected the downloaded citation data to ensure that no duplicates were included and that no articles were accidentally missed. These data were exported to two separate Comma Separated Values (CSV) files, one for each of the areas of interest. The titles of the publications from each body of literature were then merged with their respective abstracts. Our final datasets for further analysis comprised the titles and abstracts of 4,827 articles in the environmental sciences, and the titles and abstracts of 2,671 articles in the business/management literature (total of 7,498 records). Table 1 summarizes the sources and years of publication for the articles.

**Table 1:** Articles included in the analysis.

**Environmental Journals**					
Year	Annual Review of Environment and Resources	Global Environmental Change	Nature Climate Change	Wiley Interdisciplinary Reviews: Climate Change	Total
2011	18	138	182	63	401
2012	20	88	276	43	427
2013	19	166	282	44	511
2014	23	275	322	58	678
2015	18	148	317	45	528
2016	22	114	283	57	476
2017	26	111	234	54	425
2018	21	127	290	52	490
2019	17	119	286	55	477
2020	0	98	258	58	414
Total	184	1,384	2,730	529	4,827
**Management Journals**					
Year	Academy of Management Journal	Academy of Management Review	Journal of International Business Studies	Journal of Management	Total
2011	62	36	63	68	229
2012	66	36	44	63	209
2013	80	43	49	73	245
2014	78	32	56	84	250
2015	79	38	56	79	252
2016	95	43	58	82	278
2017	92	39	62	83	276
2018	93	45	62	126	326
2019	76	61	87	132	356
2020	44	26	91	89	250
Total	765	399	628	879	2,671

### 3.1 Entity linking

We proceeded with analyzing key research topics emerging from the titles and abstracts of the academic articles using an entity linker. An entity linking system uses algorithmic technology to identify meaningful text sequences (here referred to as ‘topics’) in the unstructured abstract text and title of each article and assigns unambiguous identifiers to them. Entity linkers can recognize and merge strings that describe the same concept, such as the U.S., USA and the United States of America [21]. Entity linking tools follow three steps. First, the tool identifies a list of possible topics, sometimes also referred to as mentions. Second, these topics are then disambiguated by linking them to a large catalogue of text. The disambiguation is an essential step as words often have multiple meanings–for example, the term “Mercury” can refer to the chemical element, the planet, or the Roman god [22], but is also used as company name, in the context of computing, and even for fictional characters and places in films and literature. The disambiguation step analyses the context in which the term is used to then label identified text segments using designated tags based on information gathered from the catalogue of text, for instance, to decide on “Mercury (planet)”. Lastly, the algorithm assigns scores to each possible topic to reflect the relevance of the respective topic for the overall context in which the topic is used. This score can be thought of as a confidence measure. In the last stage, the algorithm removes topics that receive low confidence scores. A further comprehensive discussion of entity linking is offered by Cornolti et al. who also discuss the application of entity linking to different types of datasets [23].

Here, we used the tool TAGME for the entity linking analysis. TAGME is an entity linker that is widely available and has been viewed by other researchers as highly effective in extracting key topics from academic texts [23, 24]. Whilst TAGME was initially designed for short, unstructured texts, it has subsequently been applied to longer texts, and was deemed to be a suitable tool for annotating text segments on-the-fly and with high precision [25]. Wikipedia was selected as the catalog of choice as it offers a trade-off between a catalog with a rigorous structure but low coverage (for example, the high-quality entity catalog WordNet and CYC), and a large collection of texts with broad coverage but unstructured and noisy content (for example, the worldwide web) [26].

To carry out the various entity linking steps detailed above, we ran the analysis in Python 3 to analyze the CSV file created in the previous step. The Python code (available on Github [27]) calls on the TAGME tool [26], which executes a process that scans the text and compiles a list of all possible topics (not ranked by any importance in the first instance). To illustrate how TAGME categorizes text and assigns identifiers, we show an example from one article included in our analysis (randomly selected) in Table 2 below. Table 2 shows the input and output of the TAGME annotation process [26]. The text on the left side served as an input into the process. The topics on the left side are the outcome of entity linking system.

**Table 2:** TAGME example.

Text of the abstract	TAGME topics
“This article reviews the political economy of government choice around technology support for the development and deployment of low carbon emission energy technologies, such as Carbon Capture and Storage (CCS). It is concerned with how governments should allocate limited economic resources across abatement alternatives. In particular, it explores two inter-related questions. First, should government support focus on a narrow range of options or be distributed across many potential alternatives? Second, what criteria should be considered when determining which specific technologies to support? It presents a simple economic model with experience curves for CCS and renewable energy technologies to explore the lowest cost alternatives for meeting an emission abatement objective. It then explores a variety of economic and political factors that must be considered when governments make decisions about technology support.”	Carbon_capture_and_storage
	Economic_model
	Renewable_energy
	Decision-making
	Economic_development
	Economy
	Energy
	Energy_technology
	Government
	Low-carbon_emission
	Low-carbon_power
	Political_economy
	Technology

Citation: Torvanger & Meadowcroft [28]

Note: TAGME tends to assign duplicate tags to topics repeated in the abstract (e.g., Carbon Capture and Storage, or CCS, is mentioned multiple times in the example above, and is thus tagged as a repeated topic). We have not listed duplicate tags in this table for purposes of readability, but they are included in the statistical analyses.

To ensure that the annotation process was rigorous and precise, we applied parameter choices for TAGME that have yielded good results in previous analyses [29]. Relevant parameters in the code allow to adjust the specificities of the annotation task (for example, the length of the text window to be annotated) [29]. The parameters we adopted are long_text 10, epsilon 0.427, rho = 0.1613. The value of long_text specifies the shifting window of the text to be annotated (which can be thought of as a scanner that runs over the text). This value can range from 1 to 10 (i.e., defining a shorter versus a longer shifting window). The value of epsilon can range from 0 to 0.5 and defines whether the annotation process will favor the immediate context of the text that is analyzed (if a lower value is set) or will assign more common/general topics (if a higher value is set). The value of rho can range from 0 to 1 and is used to indicate annotations above and below a given confidence score threshold, representing the likelihood that the annotations are appropriate given their context in the input text [29]. Given that these parameters were tested in prior research [14], we are confident that the choice of parameter settings helped to reduce annotation errors while also ensuring that the output (i.e., the number of topics identified in the respective texts) was useful for the analysis.

The application of TAGME initially 7,952 identified unique topics in the environmental sciences articles, and 4,333 topics in the business/management articles. Articles can be tagged with more than one topic, for example, climate change and greenhouse gas emissions. We reviewed the tagging to remove false positives. Topics that make little meaningful sense given the context in which they were used were deleted. Examples of these include phrases such as *for but not with*, and incorrect tags such as *Trapped (Islandic TV series)*. After cleaning results, we retained 7,915 unique topics in the environmental sciences articles, and 4,293 unique topics in business/management articles.

### 3.2 Visualization of key topics

**Fig 2:**
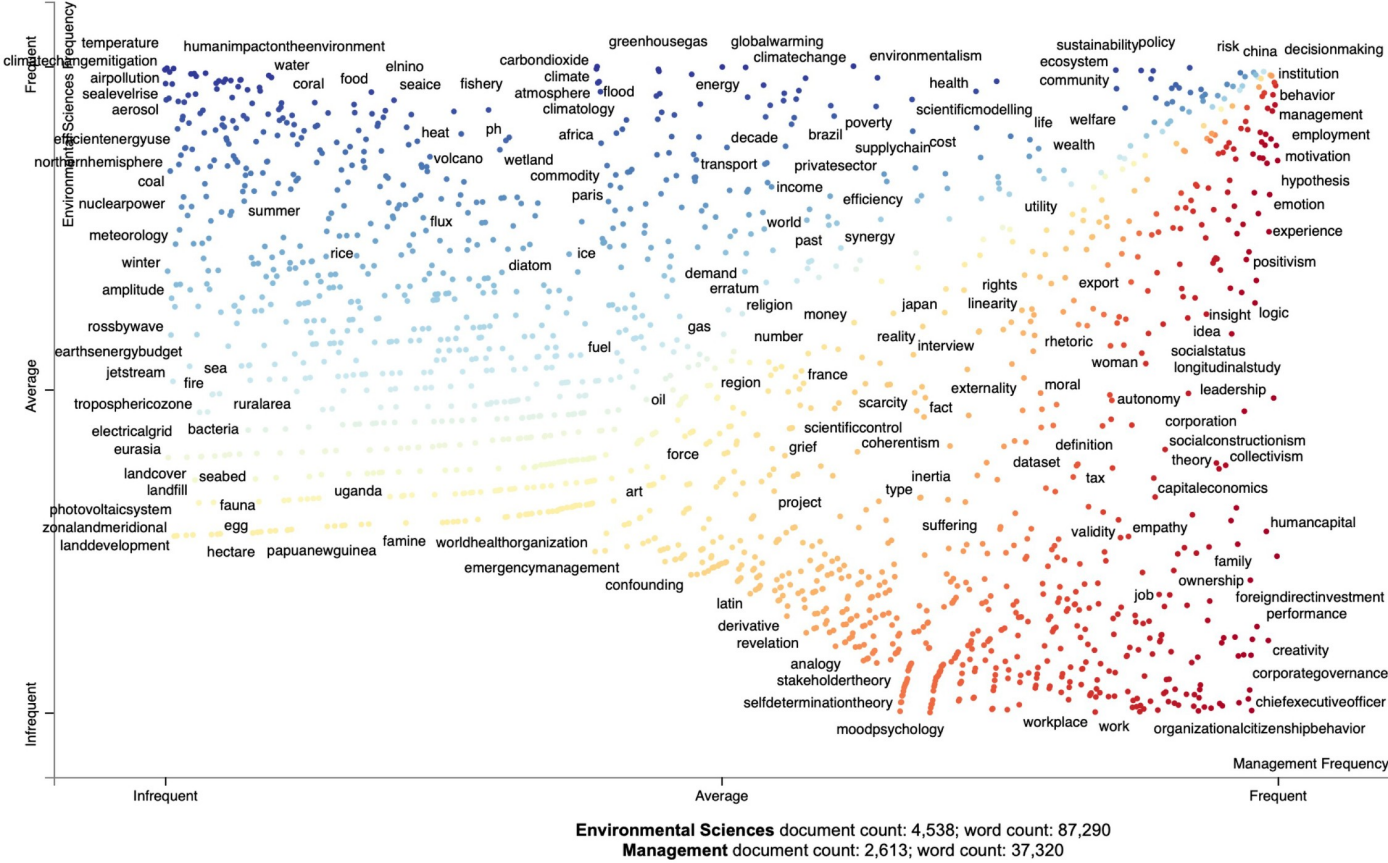
Map of common and diverging topics.

This stage of the method involves the visualization of the topics extracted by TAGME. Rather than mapping all topics, the technique here first examines them for “keyness.” The python library scattertext, used for purposes of visualizing the results in Fig 2 (below), employs a Scaled F-Score to determine the saliency of topics identified [30]. For our study, scattertext visualizes the topics within and across the business/management and environmental sciences literature to determine topics that are salient in each of the two fields, and topics that are common, or joint topics across the two fields. The term salient thereby refers to topics that are mentioned more frequently within each field (as determined by the Scaled F-Score, see above) and can thus be considered as relevant for that literature.

## 4 Results

The results of the analysis are shown in Fig 2 below. The axes correspond to the rank-frequency of a specific term [30]. In addition, Fig 2 shows association based on the color coding of topics. The topics (represented as dots) are colored either in red or blue based on their association with either the business/management or the environmental sciences literature. The higher up a dot is on the vertical axis, the more frequent it appears in environmental sciences literature. The further right a point is on the horizontal axis, the more it is frequent in business/management. In other words, topics that appear in the lower right corner are used frequently in the business/management literature (but not the environmental sciences literature), while topics that appear in the upper left corner are used frequently in the business/management literature (but not the environmental sciences literature). Topics that are more common to both literatures can be found closer to the diagonal. The top upper right points thus represent topics that are most frequently associated with both literatures.

An interactive version of Fig 2 is included in the appendix and allows for a closer interrogation of all topics beyond those displayed in the static version of Fig 2. Table 3 offers a summary of the topics most frequently associated with the environmental sciences literature only (column 1), with the business/management literature only (column 2), and with both literatures (column 3). While Fig 2 and Table 3 do not display the full citation information for specific journal articles connected to each dot, this information can be extracted from the program for purposes of further analysis.

**Table 3:** Table with topics.

Topics Environmental Sciences		Topics Management		Joint topics	
Climate	(E:1363, M:1)	Multinational Corporation	(E:3, M:368)	Knowledge	(E:337, M:317)
Air Pollution	(E:783, M:0)	Creativity	(E:2, M:238)	Globalization	(E:507, M:180)
Temperature	(E:768, M:0)	Leadership	(E:16, M:349)	Risk	(E:389, M:136)
Greenhouse Gas	(E:1190, M:4)	Human Capital	(E:11, M:190)	Economy	(E:352, M:142)
Carbon Dioxide	(E:384, M:0)	Corporate Social Responsibility	(E:3, M:148)	Technology	(E:292, M:157)
Climate Change Mitigation	(E:617, M:0)	Chief Executive Officer	(E:0, M:127)	China	(E:324, M:139)
Global Warming	(E:1134, M:5)	Performance	(E:2, M:136)	Behavior	(E:227, M:555)
Agriculture	(E:681, M:2)	Cognition	(E:38, M:238)	Institution	(E:247, M:379)
Human Impact on the Environment	(E:408, M:0)	Entrepreneurship	(E:1, M:129)	Decision-making	(E:347, M:325)
Climate Change	(E:4538, M:11)	Abusive Supervision	(E:0, M:119)	Uncertainty	(E:372, M:102)
Biodiversity	(E:559, M:0)	Human Resource Management	(E:0, M:118)	Innovation	(E:153, M:336)
Earth	(E:358, M:0)	Employment	(E:63, M:1160)	System	(E:330, M:115)
Land Use	(E:352, M:0)	Foreign Direct Investment	(E:7, M:123)	Evolution	(E:209, M:145)
Deforestation	(E:350, M:0)	Emotion	(E:48, M:267)	Social Change	(E:214, M:127)

Note: This table presents a list of topics that are mostly associated with the environmental sciences but not the business/management literature (column 1); topics that are mostly associated with the business/management literature but not the environmental sciences literature (Column 2), and topics that are most commonly associated with both literatures (Column 3). The numbers in parentheses represent the total number of appearances of the topic in the respective body of literature (E: Environmental sciences literature; M: business/management literature

### 4.1 Topics in environmental journals

As evident from Fig 2, core topics represented almost exclusively in the environmental sciences articles are related to concern about climate change. The topics “climate”, “climate change”, “global warming”, “greenhouse gas”, “carbon dioxide”, “temperature”, and “climate change mitigation” are among the most frequent topics associated with this body of literature. Articles that are tagged as belonging to these topics focus, for example, on developing cost-optimal and equitable mitigation scenarios across different countries, especially with a view of limiting global mean temperature increase below 1.5°C [31–36], specific options such as reforestation and forest-based climate mitigation [37–39], changes in consumer choices and demand [40–42], emissions pricing of food commodities [43], as well as carbon sequestration and CO_2_ capture and storage solutions [44, 45].

Attention to topics related to climate change is closely followed by attention to closely related topics, such as “human impact on the environment”, “biodiversity”, “deforestation” and “land use”. Research on these topics focuses on the adverse consequences of human impact on ecosystems, covering topics such as the negative impacts of deforestation, biodiversity and habitat loss, degradation of ecosystems and the degradation of water resources [46–50]. Several publications in this area are connected to the Planetary Boundaries framework [51–53], which tracks the risk that human activity will generate large-scale abrupt or irreversible environmental changes by interfering with nine earth system processes (biosphere integrity, land system change, freshwater use, biogeochemical flows, ocean acidification, atmospheric aerosol leadings, ozone depletion, the release of novel entities such as plastic pollution and climate change) [54].

Many articles in environmental research are tagged as belonging to more than one topic, which reflects the interconnectedness of socio-economic drivers and environmental outcomes. For example, articles within the environmental sciences field focus on Shared Socioeconomic Pathways (SSPs) and offer an the integrated analysis of future climate impacts, vulnerabilities, adaptation, and mitigation [55–62], while others examine the implications of policy decisions on future climate change outcomes [63–67], which means that they are tagged as belonging to more than one category.

### 4.2 Topics in business/management journals

Core topics represented almost exclusively in the business/management journals included in this analysis are related to firm structure and expansion, such as “multinational corporation”, “foreign direct investment”, and the role of the “Chief Executive Officer”. Articles examine, for example, how the selection and characteristics of the CEO and board impact outcome variables–such as firm performance, investment in R&D (research and development), or strategic change [68–73]. Articles interested in the impacts of internationalization focus on overseas expansion and the growth of overseas market potential, especially in emerging economies [74–76]. It should be noted here that one of the journals selected for inclusion is the Journal of International Business Studies, which traditionally features articles with an international focus, thus including a high percentage of articles with international topics.

Other common topics focus on the importance of corporate members, as evidenced by the topics “human resource management” and “human capital”. Several articles focus on specific topics such as the importance of “creativity”, “cognition”, and “performance”, Within the literature, there is also a significant focus on theoretical work related to the analysis of the behavior of individual members within organizations, such as “emotion” and “abusive supervision”. The literature is also showing an interest in corporate social responsibility, but without direct connections to the environmental sciences.

### 4.3 Joint topics

As evident from Fig 2, several topics are common to both the environmental and business/management journals. Of particular interest is the topic “decision-making”, reflecting discussions about the creation of new insights on managing environmental impacts, and the applicability of these findings to policy, organizational and other decision-makers [77, 78]. Of interest are also the topics “knowledge” and “innovation”, reflecting the endeavor in both disciplinary areas to pursue new knowledge generation and innovative ideas [79].

Research in both business/management and environmental domains has focused on the topic “China” and the role of China as one of the major developing economies–the difference is that articles in the business/management field address management challenges and economic opportunities in China [80], while the environmental articles address the role of emerging economies in climate adaptation and mitigation efforts [81]. A similar observation arises when examining the “globalization” topic–articles in the environmental sciences domain analyze the environmental implications of issues such as global population growth and socio-technological transformations [82, 83] while articles in the business/management domain analyze internationalization and globalization theories that are largely focused on managing and exploring international growth opportunities in overseas markets [84].

Of joint interest are also behavioral theories, as well as more general topics that relate to future developments, including: “risk”, “uncertainty”, and “technology”. A common topic in both fields is the concern about economic development (topic “economy”). However, a key difference here is that articles published in the environmental sciences domain analyze the economic impacts of climate change [85–88], ecosystem and resource degradation [89–91], decarbonization (e.g., through the adoption of carbon sinks as well as low carbon/zero carbon technologies) and cleantech uptake [92–94], while articles in the business/management areas focus on the economic growth through organizational (firm and industrial) activities [95], without necessarily paying attention to the environmental implications of organizational growth.

Another common theme is “institution”–related to the role of institutions. Articles in the environmental sciences domain focus on the role of institutional actors (e.g., policy actors, management agencies) in fostering or inhibiting change in social, economic and environmental systems (also related to the themes “system” and “social change”) [56]. Environmental sciences articles also focus on the role of institutional actors in the management of land resources of other commons, often with references to the work of Ostrom and other commons researchers, and outline mechanisms leading to adaptive governance and community resilience, such as cooperation and social learning [96]. Articles on institutions in the business/management domain predominantly use institutional theory as the foundation, studying a range of cultural-cognitive, normative, and regulative actors and elements that shape the development of corporate and industrial activities over time or in different cultural or international contexts [97].

## 5 Discussion

We have presented a method to map the convergence and divergence of research topics across different journals and fields to facilitate the creation and exchange of knowledge across fields. Our analysis is based on a sample of high-impact factor journals; however, the method presented here can be easily expanded to different journals, fields, and collections of articles. Below, we discuss how mapping the convergence and divergence of topics can allow for the exploration of new topic areas in interdisciplinary research.

### 5.1 Identification of areas for interdisciplinary research collaboration

Interdisciplinary Research Maps can be used to identify areas for interdisciplinary research collaboration in a number of different ways: (1) by investigating topics that are frequently associated with both literatures, and (2) by investigating topics that are frequently identified in one literature, but not the other. We use our sample of articles sourced from the top business/management and environmental sciences journals as well as the resulting map in Fig 2 as the context to discuss these options.

As evident from Fig 2, there are several areas where the fields we examine already converge, with joint interest in topics such as decision-making, the creation of knowledge, China-related research as well as globalization. These topics are frequently associated with both literatures and thus provide possible fruitful avenues for future interdisciplinary research. For example, research could explore how economic opportunities in China can be combined with the pursuit of climate adaptation and mitigation efforts. Future research can examine similarities and differences in conceptualizations of these joint topics. For example, or analysis shows a joint interest in economic development; however, articles in the business/management areas predominantly focus on economic growth, without necessarily paying attention to the environmental implications of organizational growth. Such conceptual differences do not have to be limiting factors. Understanding similarities and differences in key topics (e.g., economy, risk, uncertainty, decision-making) and realizing their importance to both fields will allow for the identification of domains and topics that represent important fundamental concepts in the development of knowledge across fields.

In addition, opportunities also exist in connecting topics across literatures that are not yet connected (i.e., a topic associated predominantly with one field). For example, our mapping approach shows that a frequent topic in the environmental sciences is concern about climate change; however, this topic is virtually absent from the business and management field. A key question is why this is the case as it stands to reason that climate change has not just impacts on the natural environment, but also on economic entities such as firms and industries. Some articles have started to notice this disconnect and are urging for further interdisciplinary research [98]. Vice versa, key management decisions (e.g., maintaining profitability) are not a core topic represented in the environmental sciences. Nonetheless, there are opportunities here to bridge such divergent topics. Some recent articles (e.g. in Nature Climate Change and Global Environmental Change) have started to engage with questions about how climate change impacts business and management decisions, including asset valuations and investment decisions [99, 100]. Future opportunities exist to broaden research at the intersection of the two fields.

### 5.2 Limitations

As with every approach, there are also limitations with the approach presented here. First, entity linking is still a new approach and will require further robustness testing across different contexts and research settings. While it has found applications in understanding topic evolution within a particular field of research, there are undoubtedly opportunity to fine-tune the approach for interdisciplinary settings, in particular to ensure that the entity linking approach correctly tags topics in very specialized research areas. One opportunity for further analysis is to examine improvements in natural language processing, and to use those to undertake further refinements to improve the quality of tagging and entity linking to overcome possible limitations. Future research can also investigate the use of a different reference catalogue.

Second, we have only used a sample of articles and journals to illustrate our analysis here–we recognize that a focus on high impact factors has limitations; however, the analysis here was aimed at a first attempt to illustrate the application of Interdisciplinary Research Maps to visualize different knowledge domains, rather than an attempt to map and review all available literature on the topic. Future research applying Interdisciplinary Research Maps can overcome such limitations by considering other sampling techniques.

## 6 Conclusion

Without access to robust time‐saving tools and visualizations, researchers will be challenged to synthesize research outputs effectively [3]. Our approach offers a first attempt at visualizing conceptual commonalities and differences across two different fields of research. We suggest that interdisciplinary research endeavors can focus on (1) investigating topics that are common across fields and to develop joint research endeavors at this interaction, or (2) on investigating topics that are frequently associated with only one field to see if these topics could be meaningfully integrated into other research fields. Future work can build upon the tool presented here to develop meaningful work that sits at the boundaries of science, business, ethics and politics, and bridges different disciplinary areas.

## Supporting information

S1 File (HTML)
